# Supplementary material for: Response strategies to acute and chronic environmental stress in the arctic breeding Lapland longspur (Calcarius lapponicus)
Source: Commun Biol. 2024 Dec 19;7:1654. doi: 10.1038/s42003-024-07370-4 (PMC11659517; doi:10.1038/s42003-024-07370-4)
Supplement: Supplementary file 1 — Supplementary information [file 42003_2024_7370_MOESM1_ESM.pdf]

Supplementary Figures

Response strategies to acute and chronic environmental stress in the arctic breeding Lapland longspur (*Calcarius lapponicus*)

Zhou Wu* *et al.*

*Corresponding author. Email: zhou.wu@roslin.ed.ac.uk

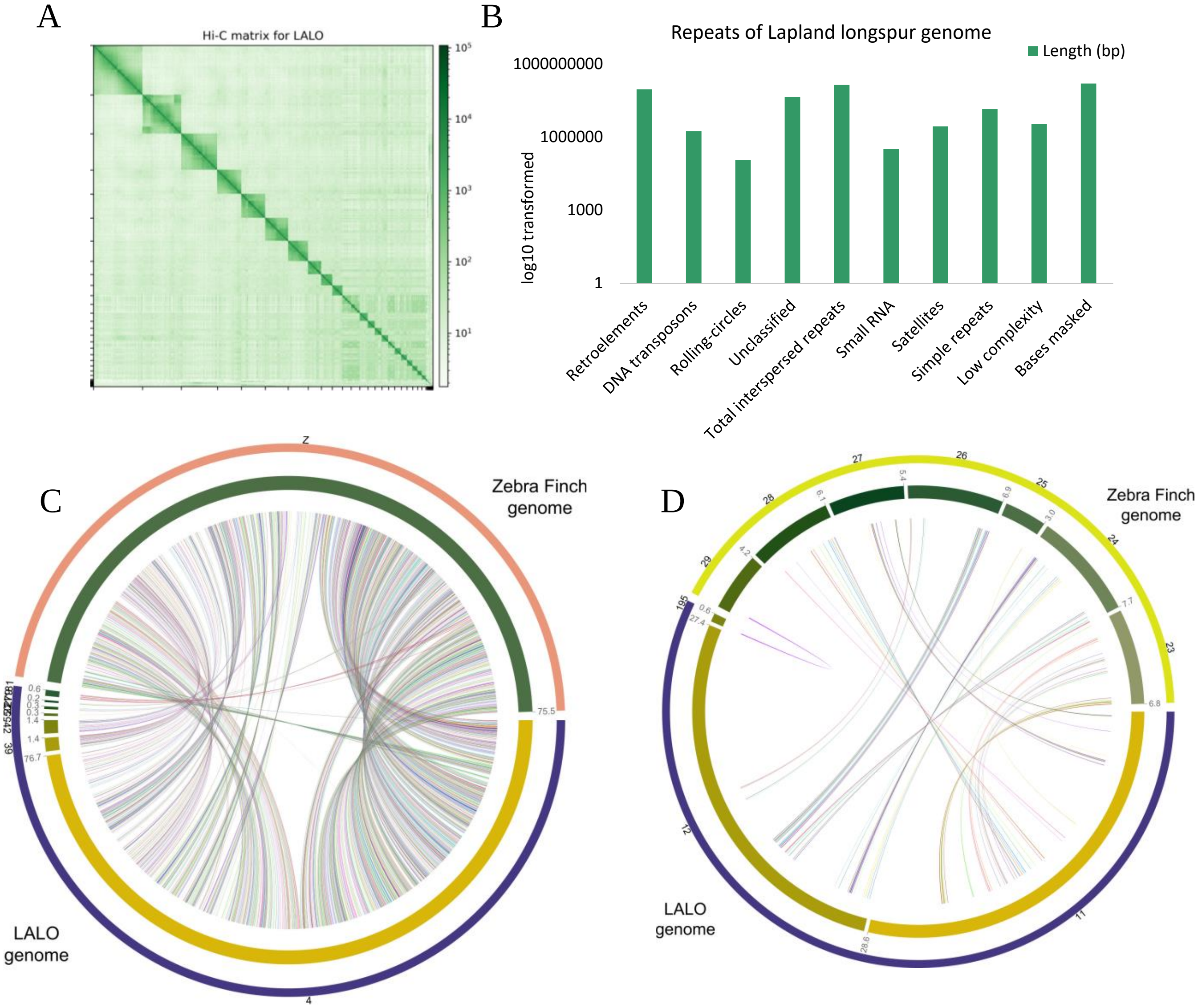

Fig. S1. Genome assembly of Lapland longspur (*C. lapponicus*). (**A**) The interaction matrix shows the Omni-C results for the genome assembly. (**B**) Repeat content of the Lapland longspur genome. The x axis denotes the repeat category, the y axis denotes the length of the repeat elements (log10 transformed). (**C**) Alignment between zebra finch chromosomes and Lapland longspur scaffolds for Z chromosome and (**D**) micro-chromosomes.

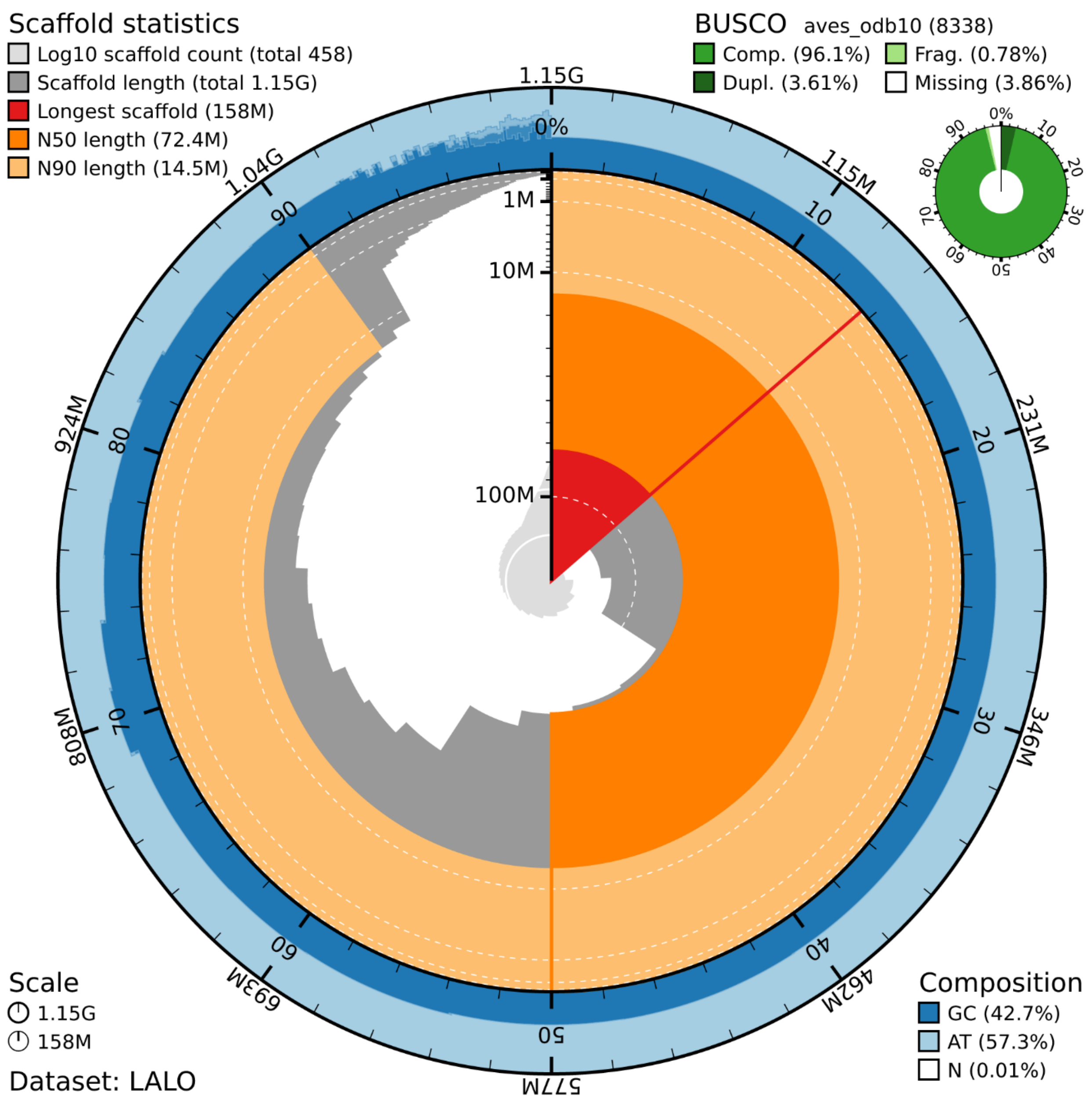

Fig. S2. Assessment of the Lapland longspur genome assembly. The snail plot presents the key scaffold statistics of the assembly, including total length, GC content, and scaffold N50 value. The BUSCO score, assessed using the *aves_odb10* database, is included in the upper right corner of the figure.

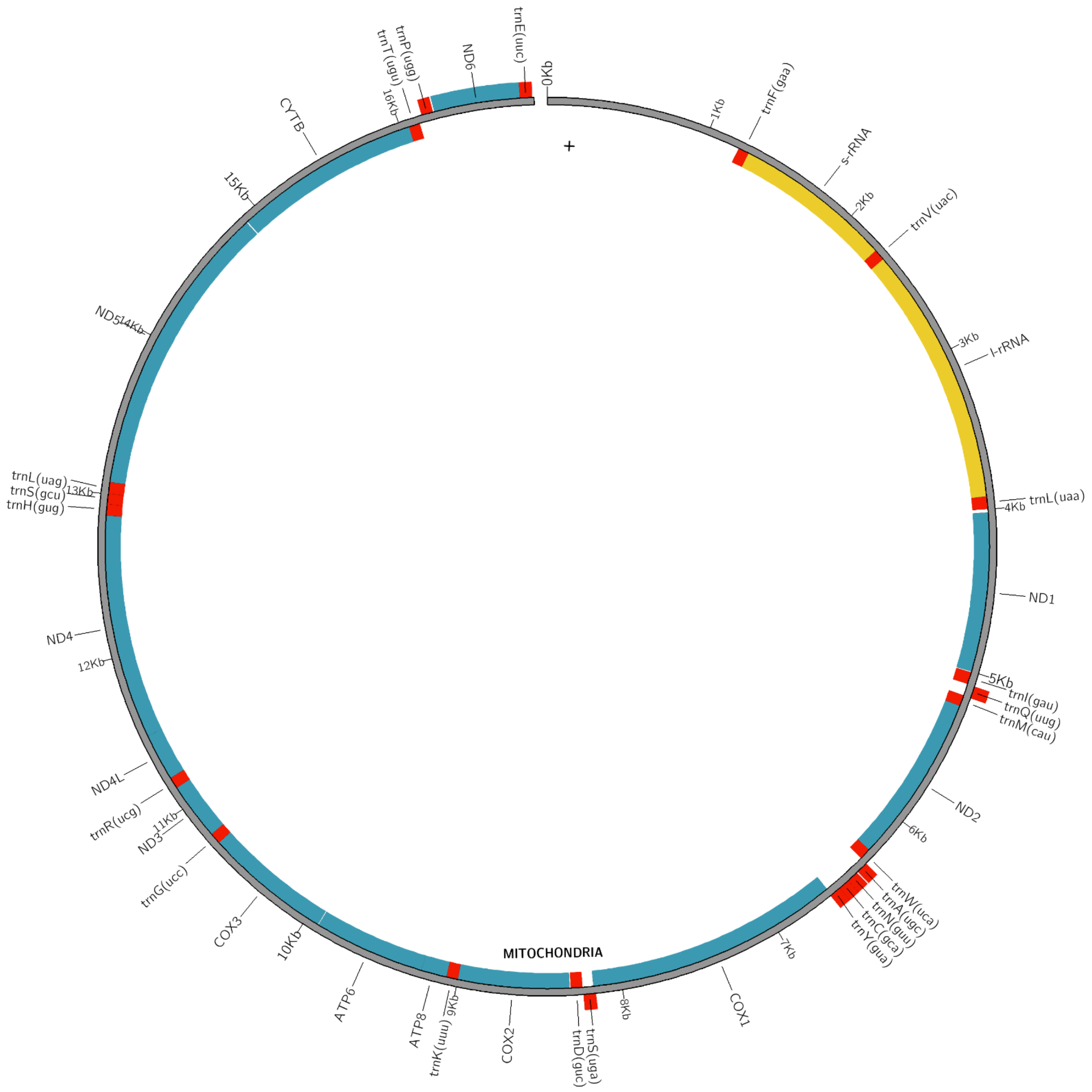

Fig. S3. **Mitochondrial genome and gene annotation of the Lapland longspur genome.**

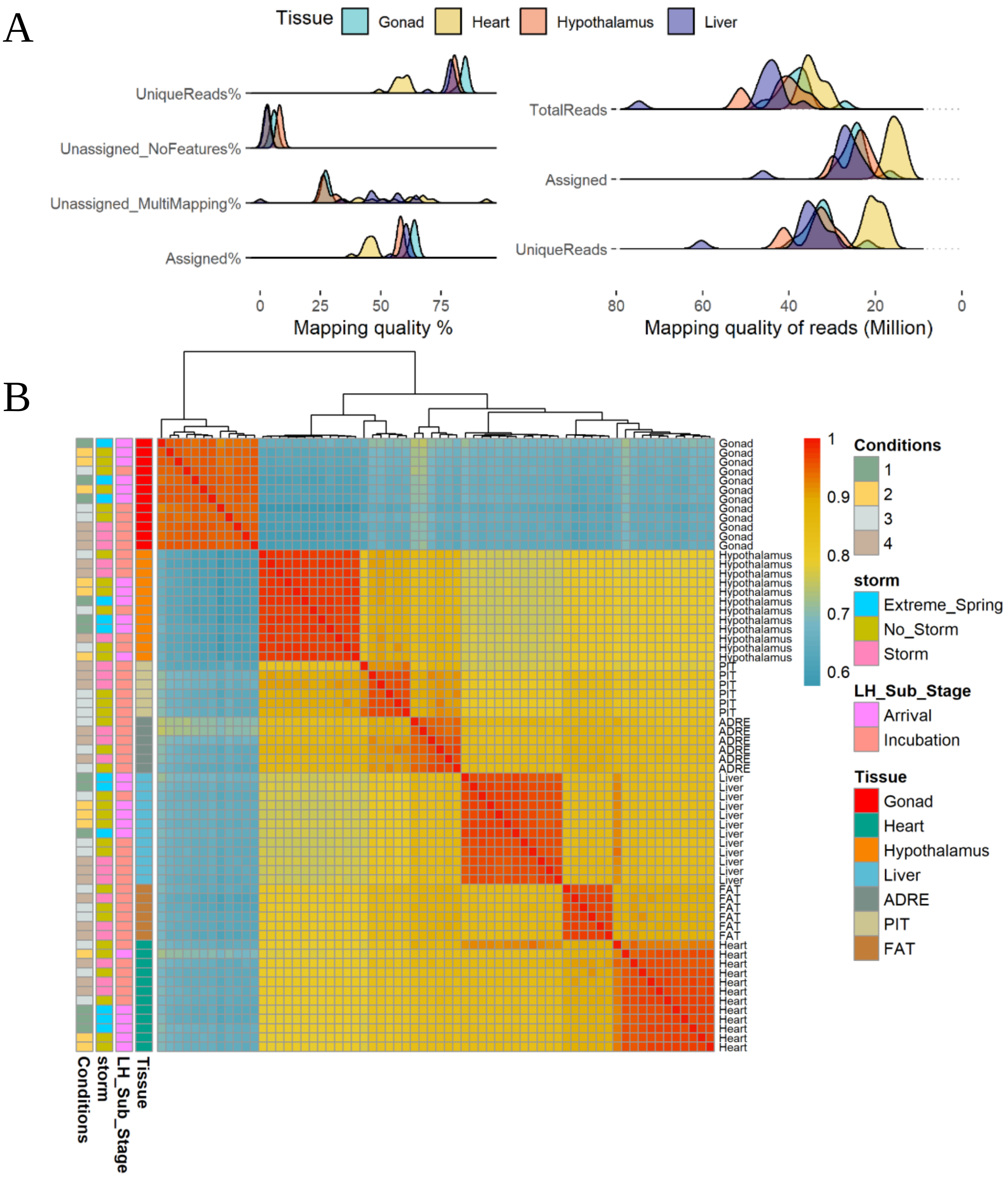

Fig. S4. Expression profile of RNA-seq data. (**A**) RNA mapping quality summarised by tissue. (**B**) Heatmap shows the correlation between all RNA-seq samples, demonstrating a tissue-specific pattern. The basic information for each sample is displayed on the right.

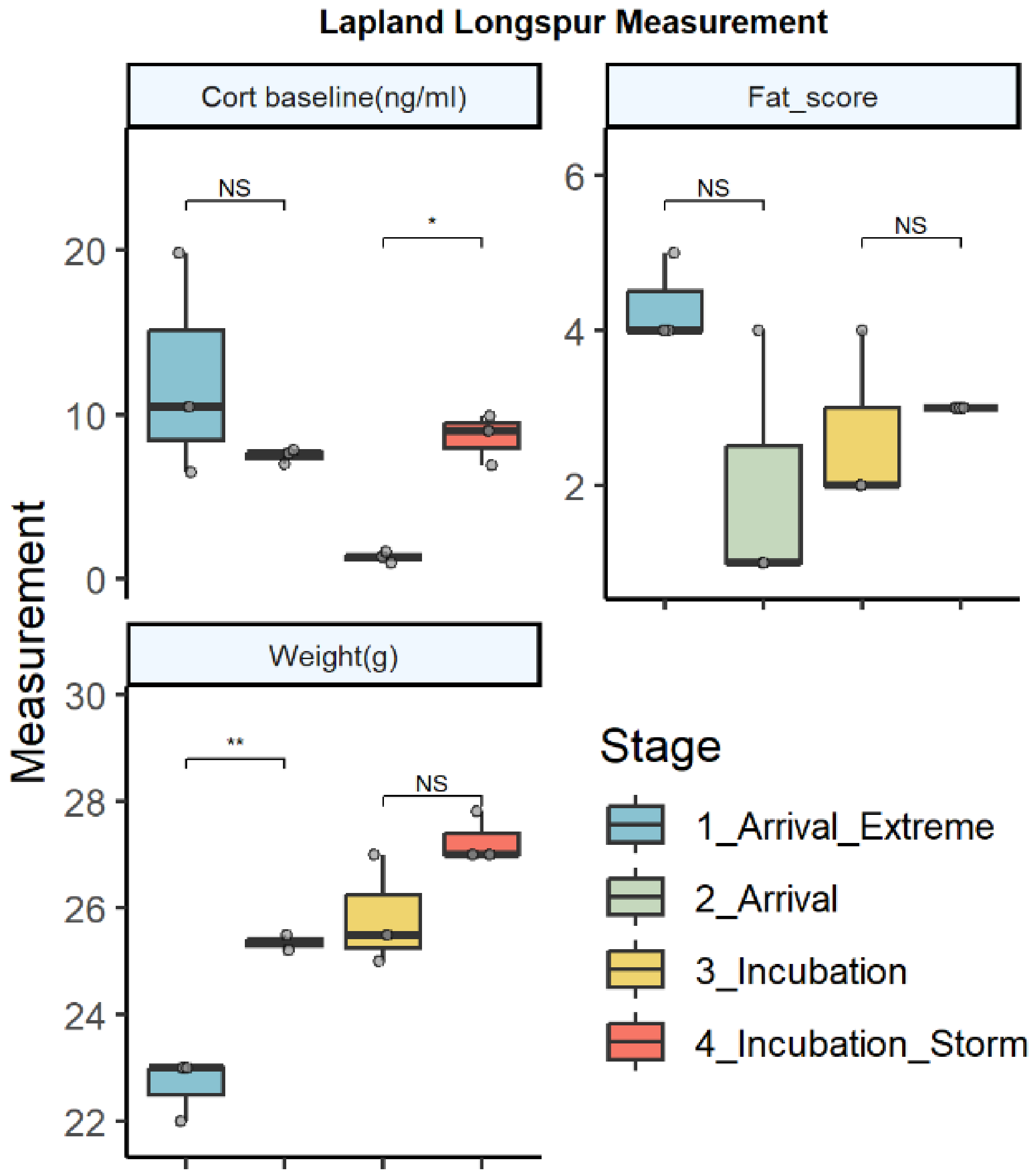

Fig. S5. Phenotypic measurement of Lapland longspur in the four studied conditions. The four environmental conditions are at two life-history stages (arrival and incubation) and are (1) 2013 arrival - extreme spring, (2) 2016 arrival - spring and storm free, (3) 2016 incubation - storm free, (4) 2016 incubation - snowstorm. Corticosterone is abbreviated as cort. NS = not significant; * = P<0.05; ** = P<0.01.

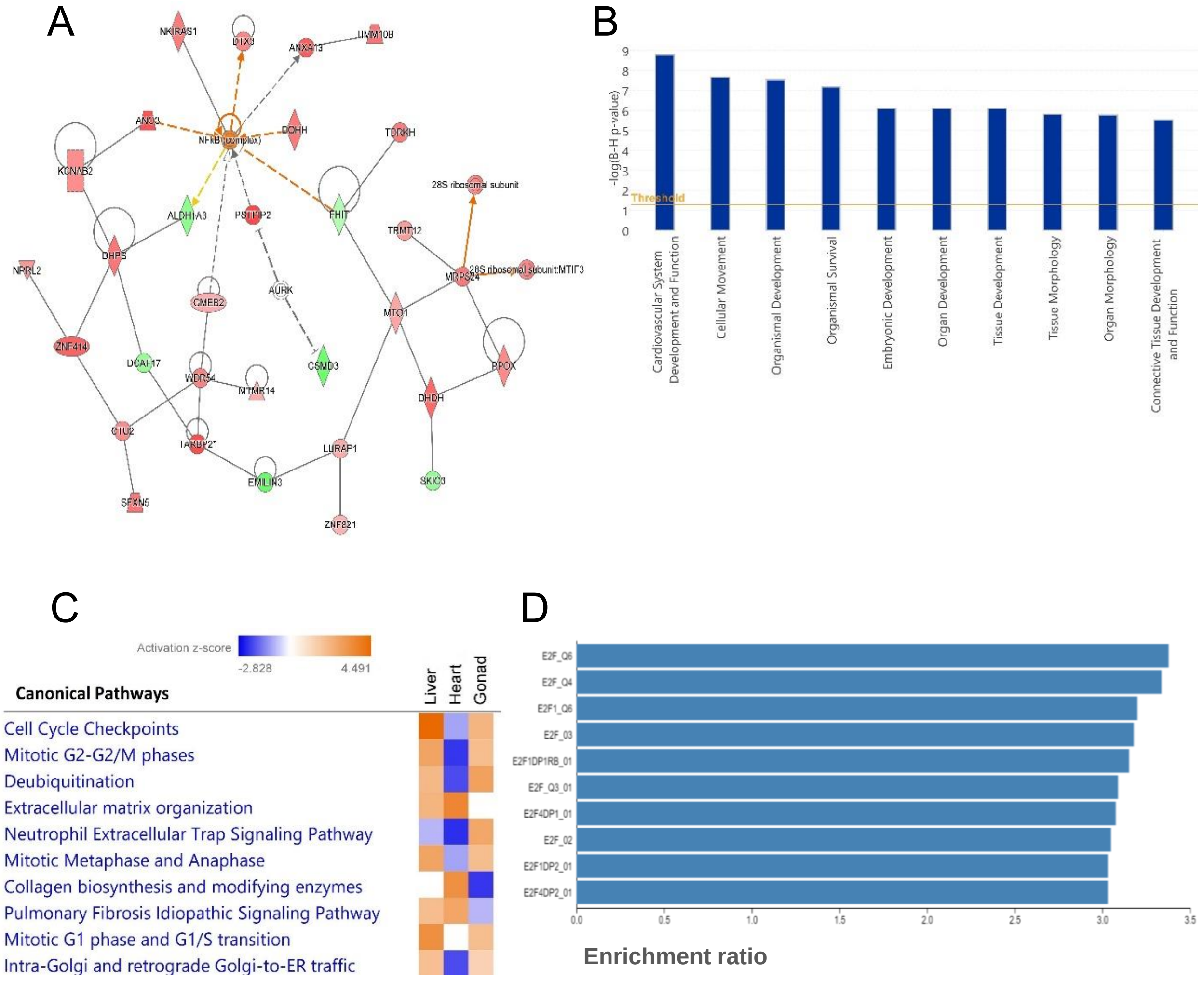

Fig. S6. Enriched pathways, networks and transcription factor binding sites of genes differentially expressed under normal conditions. (**A**) Significant network in gonad was identified using DEGs detected between benign life history stages (LHS). (**B**) Top 10 enriched molecular functions detected in heart (benign LHS). (**C**) Overview of significant canonical pathways across three tissues (benign LHS). (**D**) The significant transcription factor targets that are overrepresented in the liver (benign LHS).

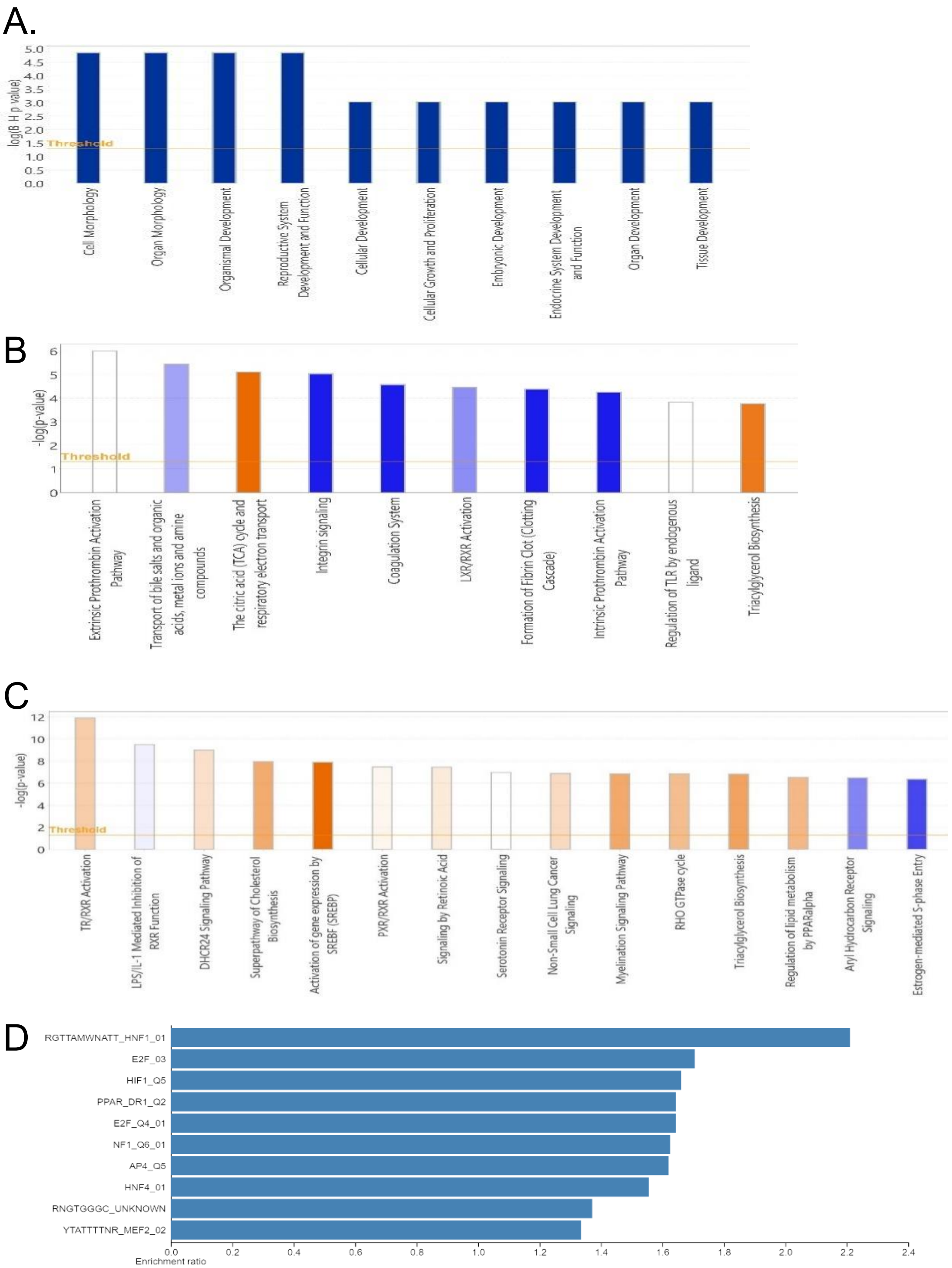

Fig. S7. Enriched pathways and transcription factor binding sites of genes differentially expressed under extreme conditions. (**A**) Top 10 molecular functions detected in gonad (extreme spring). (**B**) Most significant pathways detected in the liver (extreme spring). (**C**) Most significant pathways detected in liver (snowstorm). (**D**) The significant transcription factor targets that are overrepresented in liver (snowstorm).

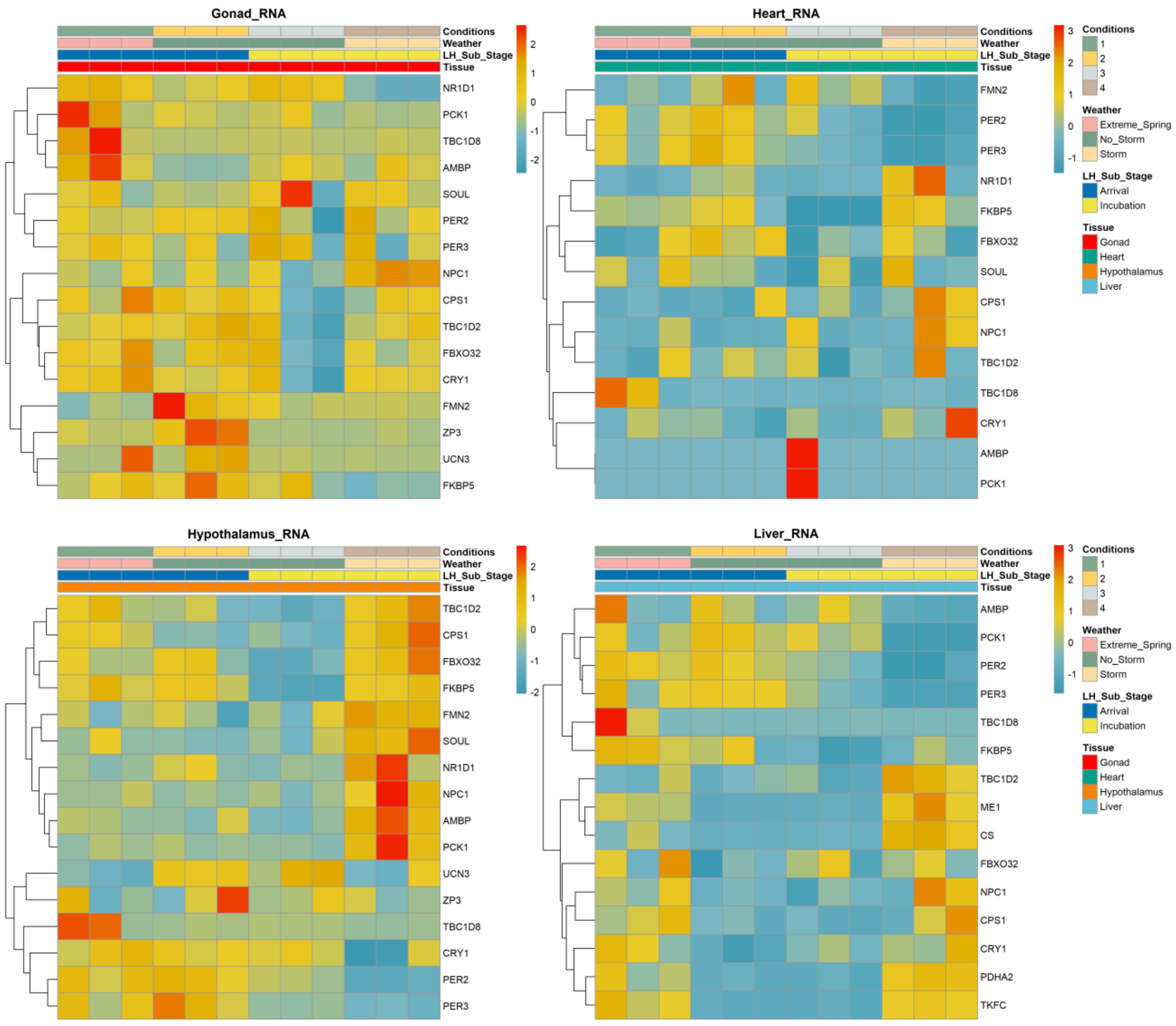

Fig. S8. Expression of genes of interest in each of the four tissues tested in the two extreme weather events.

Tissues are shown in the title of each grid, with each row showing a gene of interest, and each column showing an individual. The colour of the cell shows the relative expression level. The weather condition and the life-history stages of individuals are shown by colours at the top of each grid.

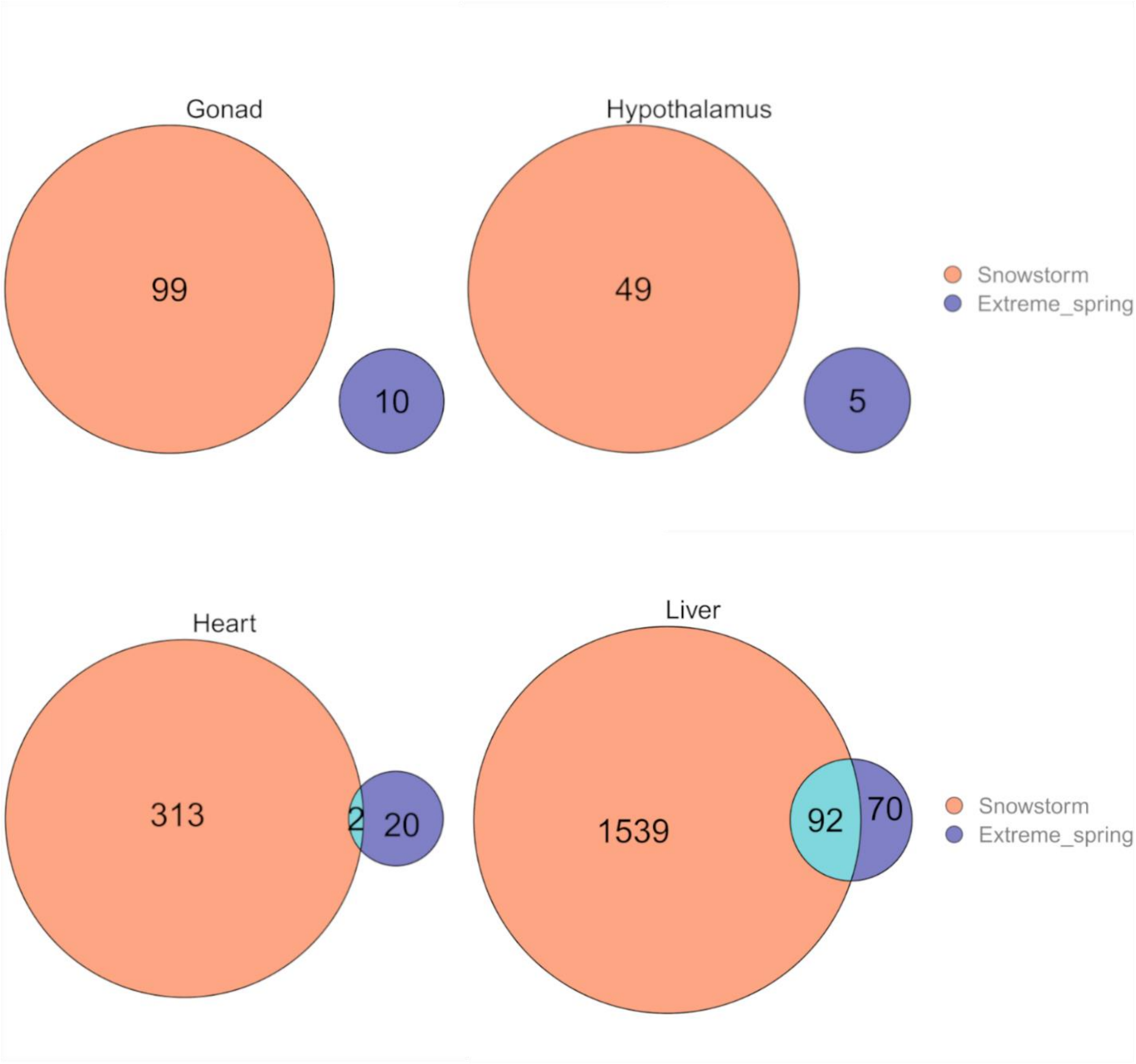

Fig. S9. Comparison of the tissue-specific differentially expressed genes.

Venn diagrams show the DEGs between the two extreme weather conditions in each tissue.

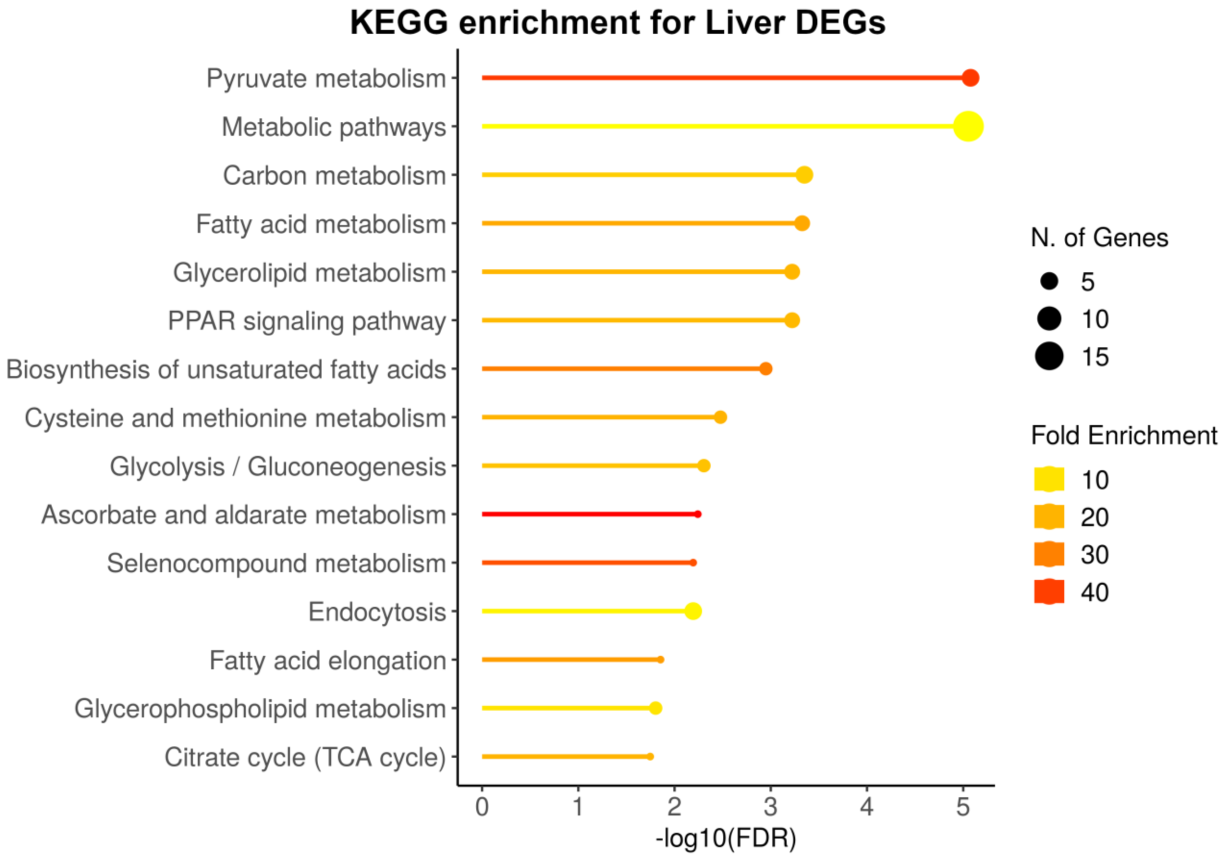

Fig. S10. **Function of Differentially Expressed Genes in liver.** KEGG pathway enrichment for liver DEGs that were identified in both extreme weather events (i.e., the extreme spring and the snowstorm).

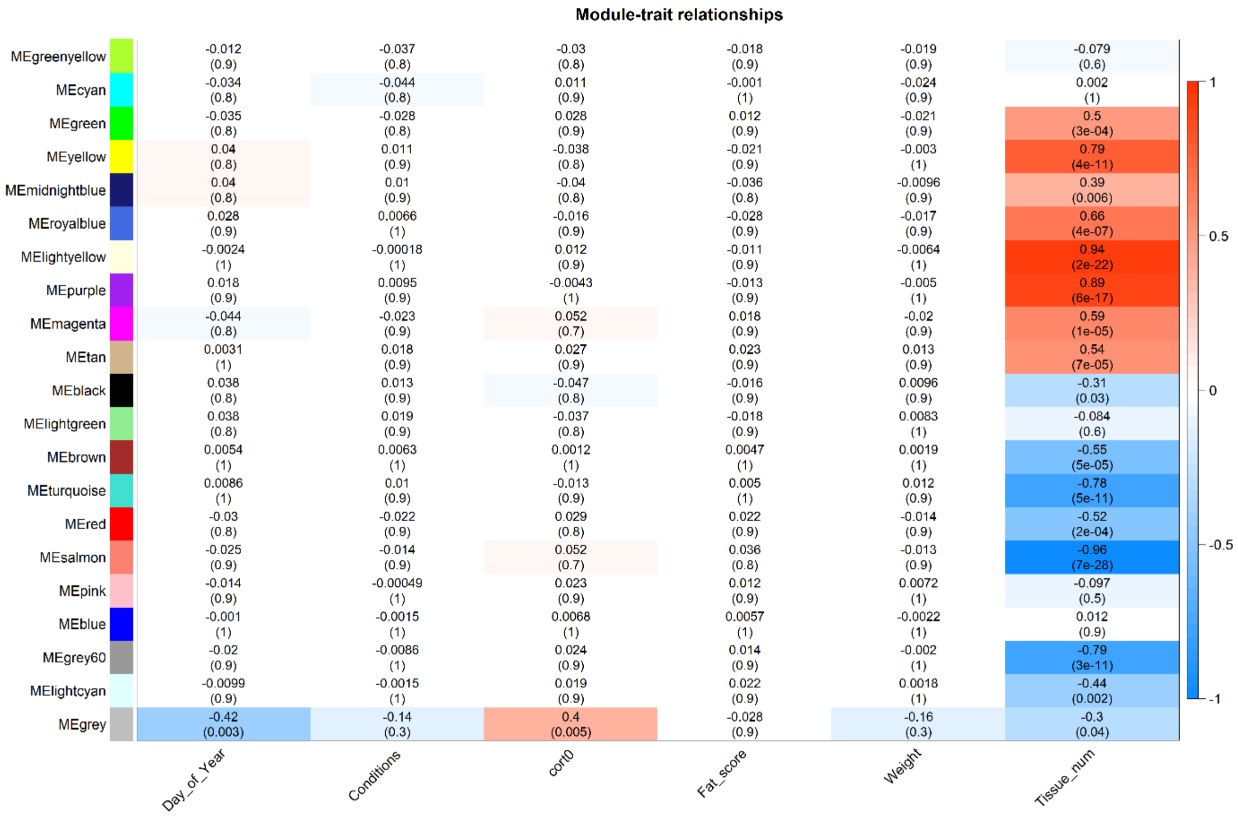

Fig. S11. WGCNA results of gene network and correlation with phenotypic data. Modules of genes identified and their association with traits. The correlation coefficients between the modules and traits with the P-value in parentheses are shown in each cell (red - positive correlation, blue - negative correlation). Traits are labelled in the text at the bottom. The cort0 denotes the baseline corticosterone level (ng/ml).

Supplementary Tables

Table S1. Repeat content of Lapland longspur genome assembly.

Repeats	Count	Length (bp)	Percentage (%)
Retro-elements	233,971	90,082,761	7.8
DNA transposons	7,139	1,739,421	0.15
Rolling-circles	267	111,322	0.01
Unclassified	66,923	43,335,542	3.75
Total interspersed repeats		135,157,724	11.7
Small RNA	952	313,698	0.03
Satellites	3,981	2,712,556	0.23
Simple repeats	249,465	13,799,903	1.19
Low complexity	51,084	3,330,926	0.29
Bases masked		1,55,365,615	13.45

Table S2. Differentially expressed genes (DEGs) detected across all tissues and weather conditions. See the separate file for Supplementary Table S2.

Table S3. Chromosome assignment for the Lapland longspur assembly.

LALO_scaffold	Chr	Length(bp)
Scaffold_2_122311246	1	122,311,246
Scaffold_5_75446533	1A	75,446,533
Scaffold_1_157814153	2	157,814,153
Scaffold_3_115401765	3	115,401,765
Scaffold_6_72367164	4	72,367,164
Scaffold_18_20846563	4A	20,846,563
Scaffold_7_64436748	5	64,436,748
Scaffold_9_35214236	6	35,214,236
Scaffold_8_40764762	7	40,764,762
Scaffold_10_33079868	8	33,079,868
Scaffold_13_26301727	9	26,301,727
Scaffold_16_21459849	10	21,459,849
Scaffold_15_22068624	11	22,068,624
Scaffold_14_22655033	12	22,655,033
Scaffold_19_18855686	13	18,855,686
Scaffold_20_17779475	14	17,779,475
Scaffold_22_14454378	15	14,454,378
Scaffold_23_12556395	18	12,556,395
Scaffold_21_15934098	20	15,934,098
Scaffold_4_76689810	Z	76,689,810
MT_16827	MT	16,827
