# Supplementary material for: Response strategies to acute and chronic environmental stress in the arctic breeding Lapland longspur (Calcarius lapponicus)
Source: Commun Biol. 2024 Dec 19;7:1654. doi: 10.1038/s42003-024-07370-4 (PMC11659517; doi:10.1038/s42003-024-07370-4)
Supplement: Supplementary file 3 — Reporting summary [file 42003_2024_7370_MOESM3_ESM.pdf]

nature portfolio

Corresponding author(s): Zhou Wu

Last updated by author(s): Aug 13, 2024

Reporting Summary

Nature Portfolio wishes to improve the reproducibility of the work that we publish. This form provides structure for consistency and transparency in reporting. For further information on Nature Portfolio policies, see our Editorial Policies and the Editorial Policy Checklist.

Statistics

For all statistical analyses, confirm that the following items are present in the figure legend, table legend, main text, or Methods section.

n/a	Confirmed	
☐	☒	The exact sample size (*n*) for each experimental group/condition, given as a discrete number and unit of measurement
☐	☒	A statement on whether measurements were taken from distinct samples or whether the same sample was measured repeatedly
☐	☒	The statistical test(s) used AND whether they are one- or two-sided<br>*Only common tests should be described solely by name; describe more complex techniques in the Methods section.*
☒	☐	A description of all covariates tested
☐	☒	A description of any assumptions or corrections, such as tests of normality and adjustment for multiple comparisons
☐	☒	A full description of the statistical parameters including central tendency (e.g. means) or other basic estimates (e.g. regression coefficient) AND variation (e.g. standard deviation) or associated estimates of uncertainty (e.g. confidence intervals)
☐	☒	For null hypothesis testing, the test statistic (e.g. *F*, *t*, *r*) with confidence intervals, effect sizes, degrees of freedom and *P* value noted<br>*Give P values as exact values whenever suitable.*
☒	☐	For Bayesian analysis, information on the choice of priors and Markov chain Monte Carlo settings
☐	☒	For hierarchical and complex designs, identification of the appropriate level for tests and full reporting of outcomes
☐	☒	Estimates of effect sizes (e.g. Cohen's *d*, Pearson's *r*), indicating how they were calculated

Our web collection on statistics for biologists contains articles on many of the points above.

Software and code

Policy information about availability of computer code

Data collection	No software was used to collect data.
Data analysis	Genome assembly: Dovetail Genomics (California, USA) according to their standard genome assembly pipeline (https://dovetailgenomics.com/); Wtdbg2; The HiRise pipeline. Removal of duplication: Purge_Dups (1.2.5). Repeat elements: Repeatmodeler (v2.0.2) and RepeatMasker (v4.1.2). Mitochondrial genome: MitoZ software (2.2). Gene annotation: Portcullis (v1.2.0); PsiCLASS (v1.0.2); CPC2 (version 0.1).<br>RNA-seq reads trimming: Trimmomatic (0.39). Quality control: FastQC (v0.11.7). RNA-seq mapping: STAR (v2.7.8a_2021-03-08). DEGs: DESeq2 (1.34.0); edgeR (3.36.0).<br>Gene functional enrichment: shinygo 0.77; Ingenuity Pathway Analysis (IPA) software (QIAGEN Inc., https://digitalinsights.qiagen.com/IPA); WebGestalt (2023) (http://www.webgestalt.org/). Network: GeneMANIA (3.6.0).<br>The R code used to generate the figures can be accessed in the github repository: https://github.com/wzuhou/LALO_Plot.

For manuscripts utilizing custom algorithms or software that are central to the research but not yet described in published literature, software must be made available to editors and reviewers. We strongly encourage code deposition in a community repository (e.g. GitHub). See the Nature Portfolio guidelines for submitting code & software for further information.

Data

Policy information about availability of data

All manuscripts must include a data availability statement. This statement should provide the following information, where applicable:

- Accession codes, unique identifiers, or web links for publicly available datasets
- A description of any restrictions on data availability
- For clinical datasets or third party data, please ensure that the statement adheres to our policy

Sequence data presented in this paper have been submitted to the NCBI public database under the following accession numbers: Calcarius lapponicus genome assembly - JBBFKN01 (GenBank accession: GCA_039654755.1); RNA-seq data - PRJNA1023066; Iso-seq data - SRR21856897, SRR21856898, SRR21856899.

Research involving human participants, their data, or biological material

Policy information about studies with human participants or human data. See also policy information about sex, gender (identity/presentation), and sexual orientation and race, ethnicity and racism.

Reporting on sex and gender	*Use the terms sex (biological attribute) and gender (shaped by social and cultural circumstances) carefully in order to avoid confusing both terms. Indicate if findings apply to only one sex or gender; describe whether sex and gender were considered in study design; whether sex and/or gender was determined based on self-reporting or assigned and methods used. Provide in the source data disaggregated sex and gender data, where this information has been collected, and if consent has been obtained for sharing of individual-level data; provide overall numbers in this Reporting Summary. Please state if this information has not been collected. Report sex- and gender-based analyses where performed, justify reasons for lack of sex- and gender-based analysis.*
Reporting on race, ethnicity, or other socially relevant groupings	*Please specify the socially constructed or socially relevant categorization variable(s) used in your manuscript and explain why they were used. Please note that such variables should not be used as proxies for other socially constructed/relevant variables (for example, race or ethnicity should not be used as a proxy for socioeconomic status). Provide clear definitions of the relevant terms used, how they were provided (by the participants/respondents, the researchers, or third parties), and the method(s) used to classify people into the different categories (e.g. self-report, census or administrative data, social media data, etc.) Please provide details about how you controlled for confounding variables in your analyses.*
Population characteristics	*Describe the covariate-relevant population characteristics of the human research participants (e.g. age, genotypic information, past and current diagnosis and treatment categories). If you filled out the behavioural & social sciences study design questions and have nothing to add here, write "See above."*
Recruitment	*Describe how participants were recruited. Outline any potential self-selection bias or other biases that may be present and how these are likely to impact results.*
Ethics oversight	*Identify the organization(s) that approved the study protocol.*

Note that full information on the approval of the study protocol must also be provided in the manuscript.

Field-specific reporting

Please select the one below that is the best fit for your research. If you are not sure, read the appropriate sections before making your selection.

☐ Life sciences ☐ Behavioural & social sciences ☒ Ecological, evolutionary & environmental sciences

For a reference copy of the document with all sections, see nature.com/documents/nr-reporting-summary-flat.pdf

Ecological, evolutionary & environmental sciences study design

All studies must disclose on these points even when the disclosure is negative.

Study description	To study the coping strategies associated with emergency life-history stages activation in Lapland longspurs following severe inclement weather, we generated RNA sequencing dataset collected across different tissues to identify differentially expressed genes in three environmental or life history comparisons, namely (i) birds arriving in their breeding grounds during an extreme spring (May 2013) compared to a normal spring arrival (May 2016); (ii) birds incubating during a snowstorm (5th June 2016) compared with storm-free incubation (17th June 2016), for which we further examined the pituitary gland, adrenal gland, and fat tissue; (iii) birds at different annual life-cycle stages - incubation versus arrival at the breeding ground, in storm free conditions.
Research sample	Tissues from 12 male Lapland longspurs (Calcarius lapponicus) were collected across the breeding season in 2013 and 2016. Samples were collected from four breeding sites on the north slope of Alaska, USA. Birds were captured with seed-baited potter traps or mist nets. Blood samples were collected within 3 minutes of capture.
Sampling strategy	Samples collected on different weather conditions were determined by their life-history stages and the event of extreme weather

Sampling strategy	events (e.g. snowstorm). We only selected male samples because previously we showed that male birds exhibit significant changes in response to stress caused by environment.
Data collection	The birds were sedated with isoflurane and euthanized by rapid decapitation (3 min 20 s ± 52 s post capture). After euthanasia, tissues were dissected, wrapped in aluminium foil, frozen on dry ice, placed into labelled plastic bags, and kept frozen on dry ice until they were stored in a -80°C freezer upon returning the laboratory. RNA was sequenced on an Illumina NovaSeq platform. Sample collection was performed by SLM, JK, JHP, JCW. RNA extraction was done by VRB, AMAR, JSK, KM, ZW.
Timing and spatial scale	Samples were collected from four field study sites based in Alaska, USA, which included Toolik Field Station (TLFS), Galbraith Lake, Pump Station 3, and MP 297. A detailed map is included in the manuscript. Sampling date: 22/05/2013 (Arrival: Extreme-spring); 23/05/2016 (Arrival: regular spring); 17/06/2016 (Incubation: storm-free); 05/06/2016 (Incubation: snowstorm).
Data exclusions	No data were excluded.
Reproducibility	The code and software used for producing results were described in the manuscript. The R code used to generate the figures can be accessed in the github repository: https://github.com/wzuhou/LALO_Plot.
Randomization	This is not applicable to this study, as the samples used were from naturally occurring conditions. Consequently, the samples were randomly selected from the captured population.
Blinding	This is not applicable to this study, as the samples used were from naturally occurring conditions. Therefore, the grouping was determined by the life-history stages and the extreme weather events.

Did the study involve field work? ☒ Yes ☐ No

Field work, collection and transport

Field conditions	We investigated the transcriptomic response of the Lapland longspur to different environmental adversity (i.e., a chronic cold spring and an acute snowstorm), during their life history transition from pre-parental stage to incubation on the Arctic tundra of Alaska. Two life-history stages of birds and four weather conditions were included.
Location	Samples were collected from four field study sites based in Alaska, USA, which included Toolik Field Station (TLFS), Galbraith Lake, Pump Station 3, and MP 297. A detailed map is included in the manuscript.
Access & import/export	Bird tissues were kept frozen on dry ice until they were stored in a -80°C freezer upon returning the laboratory. Samples were shipped on dry ice to the Roslin Institute, University of Edinburgh, UK. The work was approved by the University of California, Davis, USA Institutional Animal Care and Use Committee (AICUC) under protocol 19758, United States Fish and Wildlife Service - Federal MB90026B-0 and The Animal Welfare and Ethical Review Body at the Roslin Institute, The University of Edinburgh, UK.
Disturbance	During sample collecting, birds were sedated with isoflurane and euthanized by rapid decapitation (3 min 20 s ± 52 s post capture) to ensure minimal disturbance.

Reporting for specific materials, systems and methods

We require information from authors about some types of materials, experimental systems and methods used in many studies. Here, indicate whether each material, system or method listed is relevant to your study. If you are not sure if a list item applies to your research, read the appropriate section before selecting a response.

Materials & experimental systems

n/a	Involved in the study	
☒	☐	Antibodies
☒	☐	Eukaryotic cell lines
☒	☐	Palaeontology and archaeology
☐	☒	Animals and other organisms
☒	☐	Clinical data
☒	☐	Dual use research of concern
☒	☐	Plants

Methods

n/a	Involved in the study	
☒	☐	ChIP-seq
☒	☐	Flow cytometry
☒	☐	MRI-based neuroimaging

Animals and other research organisms

Policy information about studies involving animals; ARRIVE guidelines recommended for reporting animal research, and Sex and Gender in Research

Laboratory animals	The study did not involve laboratory animals.
Wild animals	Tissues from 12 male Lapland longspurs (Calcarius lapponicus) were collected in 2013 and 2016. Samples were collected from four breeding sites on the north slope of Alaska, USA. Birds were captured with seed-baited potter traps or mist nets. All birds were adult and sexually dimorphic when sampled. Birds were sedated with isoflurane and euthanized by rapid decapitation. Tissues were kept frozen on dry ice until they were stored in a -80ºC freezer upon returning the laboratory. Samples were shipped on dry ice to the Roslin Institute, University of Edinburgh, UK.
Reporting on sex	All birds were adult and sexually dimorphic when sampled, therefore the sex of birds was determined by the morphological features of them. We only selected male samples because previously we showed that male birds exhibit significant changes in response to stress caused by environment (e.g., see Reference 7 and 11).
Field-collected samples	This study did not involve laboratory work with samples collected from the field.
Ethics oversight	The work was approved by the University of California, Davis, USA Institutional Animal Care and Use Committee (AICUC) under protocol 19758, United States Fish and Wildlife Service - Federal MB90026B-0 and The Animal Welfare and Ethical Review Body at the Roslin Institute, The University of Edinburgh, UK.

Note that full information on the approval of the study protocol must also be provided in the manuscript.

Plants

Seed stocks	*Report on the source of all seed stocks or other plant material used. If applicable, state the seed stock centre and catalogue number. If plant specimens were collected from the field, describe the collection location, date and sampling procedures.*
Novel plant genotypes	*Describe the methods by which all novel plant genotypes were produced. This includes those generated by transgenic approaches, gene editing, chemical/radiation-based mutagenesis and hybridization. For transgenic lines, describe the transformation method, the number of independent lines analyzed and the generation upon which experiments were performed. For gene-edited lines, describe the editor used, the endogenous sequence targeted for editing, the targeting guide RNA sequence (if applicable) and how the editor was applied.*
Authentication	*Describe any authentication procedures for each seed stock used or novel genotype generated. Describe any experiments used to assess the effect of a mutation and, where applicable, how potential secondary effects (e.g. second site T-DNA insertions, mosiacism, off-target gene editing) were examined.*
